# Prevalence of *Plasmodium* spp. in illegal gold miners in French Guiana in 2015: a hidden but critical malaria reservoir

**DOI:** 10.1186/s12936-016-1367-6

**Published:** 2016-06-09

**Authors:** Maylis Douine, Lise Musset, Florine Corlin, Stéphane Pelleau, Jérémie Pasquier, Louise Mutricy, Antoine Adenis, Felix Djossou, Paul Brousse, Frédérique Perotti, Helene Hiwat, Stephen Vreden, Magalie Demar, Mathieu Nacher

**Affiliations:** Inserm CIC 1424, Centre d’Investigation Clinique Antilles Guyane, Centre Hospitalier de Cayenne, Rue des Flamboyant, BP 6006, 97306 Cayenne Cedex, France; Equipe EA 3593, Ecosystèmes Amazoniens et Pathologie Tropicale, Université de Guyane, Cayenne, France; Laboratoire de Parasitologie, WHO Collaborating Centre for Surveillance of Anti-Malarial Drug Resistance, Centre National de Référence du paludisme, Institut Pasteur de la Guyane, Cayenne, France; Laboratoire hospitalo-universitaire de Parasitologie-Mycologie, Cayenne Hospital, Cayenne, France; Infectious and Tropical Diseases Department, Cayenne Hospital, Cayenne, France; Centres Délocalisés de Prévention et de Soins, Cayenne Hospital, Cayenne, France; Pharmacy, Centre Hospitalier de l’Ouest Guyanais, Saint Laurent du Maroni, France; Malaria Programme, Ministry of Health, Paramaribo, Suriname; Foundation of Scientific Research Suriname (SWOS), Paramaribo, Suriname

**Keywords:** Malaria, Illegal gold mining, French Guiana, Guiana Shield, Malaria reservoir

## Abstract

**Background:**

Malaria is endemic in French Guiana, an overseas territory of France on the Guiana Shield. Since 2005, notified malaria cases are decreasing. However, new data show that malaria affects many Brazilian gold miners working illegally in French Guiana, the majority of whom are not counted in official data. In addition, one major concern is the usual practice of improper self-treatment in this mining population, raising fear of the development of anti-malarial resistance. This prospective study, conducted in 2015, aimed to estimate the prevalence of *Plasmodium* spp. in illegal gold miners working in French Guiana.

**Methods:**

The recruitment of gold miners was carried out in resting sites along the French Guiana-Suriname border, where they go for supplies, medical care or leisure. After recording agreement, three malaria diagnostic methods were performed: rapid diagnostic test, microscopy and PCR.

**Results:**

Among 421 persons recruited in the study, malaria prevalence, detected by nested-PCR, was 22.3 % (CI [18.3–26.3], n = 94/421) of which 84 % were asymptomatic.

**Conclusions:**

This significant malaria reservoir in a mobile and illegal population with difficult access to a health care system raises the threat of artemisinin resistance and puts the population of the Guiana Shield at risk of new transmission foci while countries of the region aim at malaria elimination. Even though French legislation may hamper dealing with this population, France must face the reality of malaria in illegal gold miners in order to meet its commitment to malaria elimination.

## Background

With 198 million cases and 584,000 deaths in 2013, malaria is one of the most widespread parasitic illnesses in the world [1]. Malaria is endemic in French Guiana, a French overseas territory located on the Guiana Shield, between Suriname and Brazil. French Guiana is the size of Portugal, mostly covered by Amazonian forest, and populated by several different ethnic groups, including many immigrants from mainly South America or the Caribbean. Most of the 240,000 inhabitants live on the coast, but some live along the Maroni and Oyapock Rivers. The soil of this region is rich in gold. Nowadays, gold mining is the second largest industrial activity in French Guiana after the Guiana Space Centre. However, the quantity of gold that is illegally extracted every year is over five times that of the legal production [2]. Ten to 15 thousand people, mainly from Brazil, work without authorization in more than 700 different mining sites [3]. Most of these miners, also called *garimpeiros*, are in French Guiana without visas and administrative documents. This situation has dramatic ecological consequences for the forest and rivers. Living conditions in those settings are very hard with poor hygiene, exhausting work and nutritional deficiencies leading to poor health [4, 5]. Deforestation and still water favour mosquito proliferation, and notably *Anopheles darlingi,* the main malaria vector. Medical care is free in the French health centres located in the territory, but the remoteness of the mines (sometimes 4 days by boat) and the fear of law enforcement hampers effective access to care by the miners.

Malaria is endemic in French Guiana [6]. Since 2008, the generalized use of artemisinin-based combination therapy (ACT), ‘Harpie’ military operations destroying illegal mining camps and supplies, and the positive effects of malaria treatment policy in neighbouring countries, Brazil and Suriname (specially the successful programme ‘Looking for gold, finding malaria’ in mining areas in Suriname [7, 8]), contributed to a decrease in the number of cases [9]. Thus, in 2014, only 450 symptomatic malaria cases were recorded in French Guiana by passive case detection, ten times less than in 2005. [10, 11]. In French Guiana as on the Guiana Shield, the prevalence of *Plasmodium falciparum* is very high (between 30 and 45 %) comparing to the high prevalence of *Plasmodium vivax* in South America [1, 11]. Although few data are available, it seems that a large majority of malaria cases in French Guiana concern *garimpeiros* that are under-reported in official data. This is suggested by indirect observations: (1) soldiers contracting malaria after their assignment in the forest to fight illegal gold mining activities [12–15]; (2) local population outbreaks close to gold mining sites [16]; (3) gold miners diagnosed with malaria in Suriname but working in French Guiana in previous weeks; and, (4) the last three notified deaths attributed to malaria in 2013 occurred in illegal gold miners. Consequently, even if the information system used by the Health authorities gives some trends on malaria transmission in gold mines, it only covers the tip of the iceberg. In addition, one major concern, in this historical area of anti-malarial drug emergence is the usual practice of improper self-treatment with ACT in this mining population, raising fear of the development of artemisinin resistance [17].

The aim of this study was to estimate the prevalence of *Plasmodium* spp. carriers among the illegal gold-mining population working in French Guiana and the proportion of asymptomatic carriers, in order to estimate the size of the human reservoir.

## Methods

### Study sites

Assessing the malaria burden in *garimpeiros* is very difficult because of the absence or confidentiality of information on mining sites, which are mainly located in the Amazonian natural protected area. These places are often very difficult to access, leading to logistical limitations and legal issues. In addition, the climate of lawlessness and violence on these sites hampers the safe characterization of malaria transmission in these populations. However, gold miners come to the mining site in French Guiana either directly from Brazil via the Oyapock River (the border with Brazil), or from Suriname crossing the Maroni River. During their working periods, they also regularly go to ‘resting sites’ spread along these two natural borders. These sites are structured as wooden shacks built around bars/shops. They are places to rest, and to fulfil needs for supplies, medical care or leisure. This cross-sectional, multicentric, observational study was conducted between 1 January and 30 June 2015 in strategic resting sites along the Suriname–French Guiana border. It was implemented on this border only because of: (1) delays in obtaining authorization from the Brazilian Ministry of Health; (2) insufficient funding; and, (3) the majority of illegal mining activity taking place in West French Guiana.

### Recruitment

As no data were available, expected *Plasmodium* spp. prevalence was estimated at 50 % for a maximum sample size. Considering a 5 % error margin, an alpha risk of 0.05 and an estimation of a total population of 10,000 according to the French Army, a minimum of 387 subjects was needed. The sampling was achieved through chance meetings to recruit ‘seeds’ and then the ‘snowball method’ at gold-mining resting sites. Recruitment was carried out by a team with a medical doctor, a nurse and a Brazilian mediator in the resting site, once every 2 weeks during the 6-month study period. The inclusion criteria were: working on a gold mining site in French Guiana; being at the resting site for fewer than 7 days (in relation to the incubation time for malaria); being over 18 years of age; and, accepting to participate in the study. The variables collected were sex, age, country of birth, site of gold mining, presence of fever in the past 48 h, and anti-malarial treatment during the month before inclusion. After checking these inclusion criteria, informing persons and recording agreement, several diagnostic methods were used: a pin-prick blood sampling for malaria rapid diagnostic test (RDT), a drop of blood for thick and thin smears, and a 5-ml EDTA blood sample for polymerase chain reaction (PCR). If RDT was positive, the medical doctor provided immediate artemether/lumefantrine treatment according to French recommendations. An insecticide-treated net and an information flyer were also provided to each participant.

### Diagnostic methods

The RDT used was the SD Bioline^®^ Pf/Pan test (pfHRP_2_/pLDH based Standard Diagnostics), as used in health centres in French Guiana. Microscopy was performed at the Parasitology Department of Cayenne Hospital according to World Health Organization (WHO) recommendations [18]. DNA was extracted from 200 µL of whole blood with the QIAamp^®^ DNA kit (Qiagen), and a standard nested-PCR targeting the rDNA 18S was performed at the National Reference Centre of Malaria in *Institut Pasteur de la Guyane* [19]. This method has a detection threshold of one parasite/µl of blood and was therefore used as main outcome for evaluating *Plasmodium* spp. prevalence. Performances of RDT and microscopy were evaluated using nested-PCR as gold standard. Asymptomatic carriers were defined as persons having a positive PCR for malaria without any fever reported in the previous 48 h given the high prevalence of *P. falciparum* and *P. vivax* cases in the region.

### Ethical and regulatory approvals

As the recruitment took place on the Suriname border, a partnership with the Ministry of Health Malaria Programme of Suriname was implemented and authorizations obtained. In France, the study was approved by the *Comité d’Evaluation Ethique de l’Inserm*, an Ethics Committee on Research: Process No 14-187 (IRB00003888 FWA00005831). The authorization of importation of human biological samples was obtained from the French Ministry of Education and Research, Process No IE-2014-758. The database was anonymized and declared to the *Commission Nationale Informatique et Libertés*.

### Statistical analysis

Descriptive data analyses were carried out using Stata12 software.

## Results

### Study population

During the study, 421 persons working in 68 different mining sites in French Guiana were enrolled. Acceptance to take part in the study was good with a participation rate of 90.5 %, motivated mainly by the opportunity to make a free check-up or because they were grateful to receiving attention. The sex ratio male/female was 2.4 with 297 males (70.6 %). The median age was 37 years (interquartile range (IR) 30–45). The majority of the participants were from Brazil (93.8 %), 3.6 % from Suriname, 1.6 % from French Guiana, and 1 % from other countries. Thirty-eight people declared having had fever in the previous 48 h and 47 having taken an anti-malarial treatment during the past month.

### *Plasmodium* spp. carriers

The PCR prevalence of *Plasmodium* spp., the primary outcome, was 22.3 % (95 % confidence interval (CI) 18.3–26.3 %) with 94 positive persons and a majority of *P. falciparum* (47.9 %) (Table 1). The median age in the PCR-positive population was 35.5 year old (IR 28–42) and 77.7 % were male. Most people were from Brazil (n = 93/94). Among the PCR-positive people, 17 % had taken an anti-malarial treatment in the past month, and 16 % declared having had fever the past 48 h.

**Table 1:** Results of different diagnostic methods

	RDT n (%)	Thick smear n (%)	PCR n (%)
Positive rate	18 (4.3)	17 (4.1)	94 (22.3)
95 % CI	(2.3–6.2)	(2.2–5.9)	(18.3–22.3)
*Plasmodium* species			
*P. falciparum*	8 (44.4)	7 (41.2)	45 (47.9)
Presence of gametocytes		*4* (*57*)*	
*P. falciparum/P. vivax*		0	10 (10.6)
*P. vivax*	10 (55.6)	10 (58.8)	35 (37.2)
Presence of gametocytes		*8* (*80*)*	
*P. malariae*		0	3 (3.2)
*P. vivax/P. malariae*		0	1 (1.1)

* Percentages of gametocyte presence did not differ significantly between 57 and 80 % (p = 0.71)

*RDT* rapid diagnostic test; *PCR* polymerase chain reaction

The positive rate of RDT was 4.3 % (95 % CI 2.3–6.2) with 18 positive tests. Fourteen thin blood smears [positivity rate 3.3 % (95 % CI 1.6–5.1)] and 17 thick blood smears [4.1 % (95 % CI 2.2–5.9)] were positive for *Plasmodium* spp. Fifty-nine percent of the positive thick smears had *P. vivax*, of which 80 % contained gametocytes, and 41 % *P. falciparum*, of which 57 % contained gametocytes.

### Asymptomatic carriers

Asymptomatic carriers represented 84 % of the PCR-positive population, therefore a prevalence of 18.7 % (95 % CI 15–22.5) in the study population. The median age of asymptomatic carriers was 37 years old (IR 29–43).

The proportion of asymptomatic carriers was linked to the sensitivity of each diagnostic method. 53.3 % of people with positive RDT and 90.4 % of those with positive PCR were asymptomatic (Table 2). Asymptomatic carriage also varied according to the *Plasmodium* species: 84.4 % for *P. falciparum*, 88.9 % for *P. vivax*, and 60 % for *P. falciparum/P. vivax* co-infection (Table 3). Although the difference was not statistically significant (p = 0.1), mixed *P. falciparum/P. vivax* infections seemed to be more often symptomatic than mono-infections.

**Table 2:** Proportion of asymptomatic infections according to the diagnostic method

	Asymptomatic n (%)		p	p trend
	Yes	No		
RDT and PCR positive	8 (53.3)	7 (46.7)	0.003	<0.001
Thick smear and PCR positive	5 (83.3)	1 (16.7)		
PCR positive only	66 (90.4)	7 (9.6)

**Table 3:** Proportion of asymptomatic infections according to *Plasmodium* species

	Symptomatic n (%)	Asymptomatic n (%)
*P. falciparum*	7 (15.6)	38 (84.4)
*P. vivax* (includes co-infection *P. vivax/P. malariae*)	4 (11.1)	32 (88.9)
Co-infection *P. falciparum/P. vivax*	4 (40)	6 (60)
*P. malariae*	0	3 (100)

### Spatial repartition of *Plasmodium* spp. carriers

**Fig. 1:**
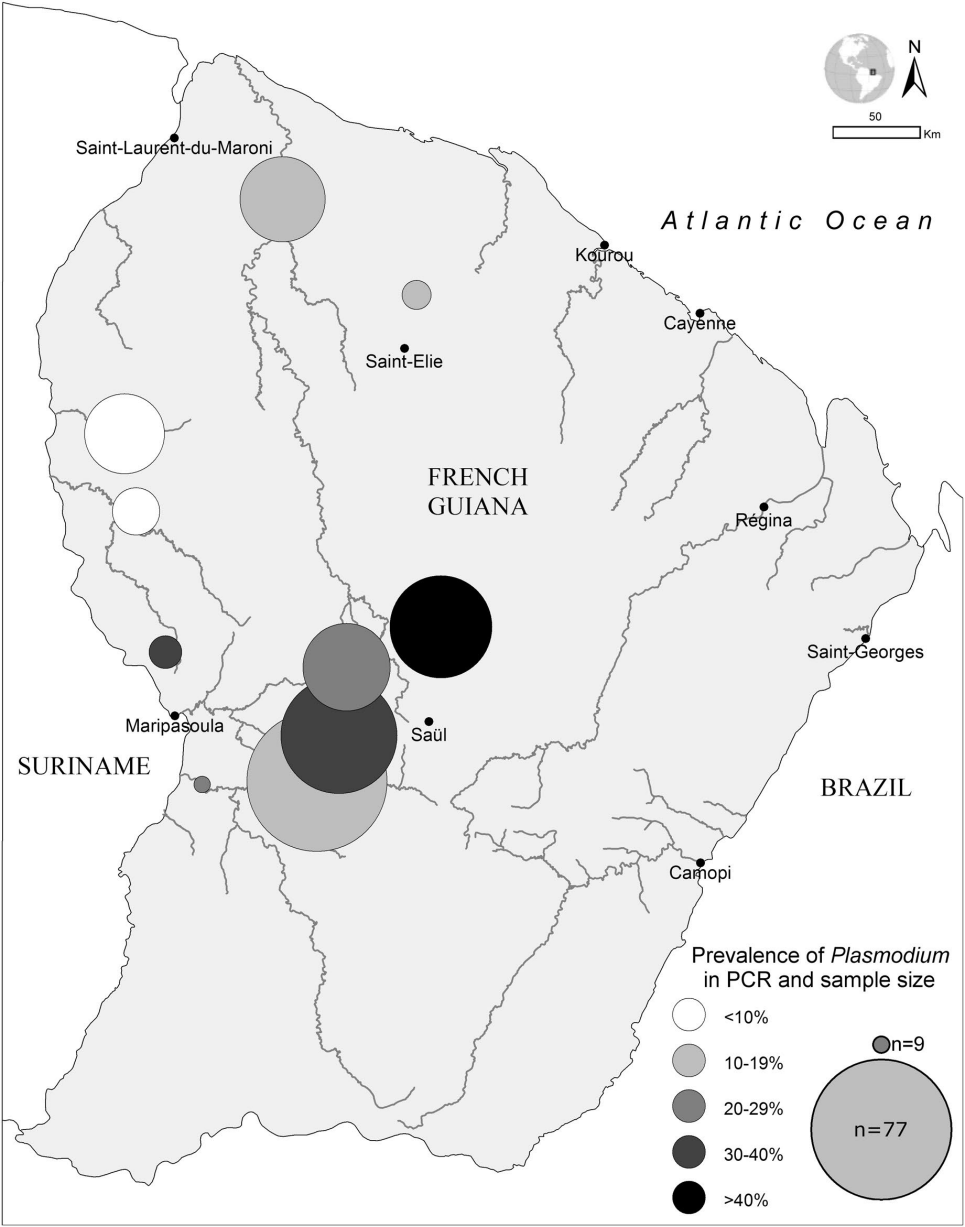
Heterogeneity of *Plasmodium* spp. carriage between the different illegal gold mining zones in French Guiana, 2015

The geographical repartition of the cases was heterogeneous among the different gold-mining areas. The 68 mining sites of origin were grouped in ten areas according to proximity and river basin. Prevalence varied from 3.8 to 46.4 % with a higher prevalence in the region located between Maripa Soula and Saül (Fig. 1). Distribution of asymptomatic persons or *Plasmodium* species did not differ between sites.

### Sensitivity and specificity of the different diagnostic tests

Compared to nested-PCR, the sensitivity of RDT was very low at 16 %, in this active case detection context, in the absence of *pfhrp2* deleted-parasites in French Guiana as observed throughout the quality control of routine diagnosis. However specificity of RDT was high at 99.1 %.

Similar results were found for microscopic examination with a sensitivity and specificity of 18.1 and 100 %, respectively. Therefore, 84 % of *Plasmodium* spp. carriers would have been missed by only using RDTs, and 82 % by only using microscopic examination. Sensitivity and specificity of those methods were higher in persons reporting fever in the past 48 h: 46.7 and 95.6 % for RDT, and 46.7 and 100 % for microscopic examination, respectively (Table 4).

**Table 4:** Performance of RDT and thick smear according to past history of fever

	RDT		Thick smear	
	Sensitivity % (CI 95 %)	Specificity % (CI 95 %)	Sensitivity % (CI 95 %)	Specificity % (CI 95 %)
Global	16 (9.9–27.7)	99.1 (97.3–99.7)	18.1 (11.6–27.1)	100 (98.8–1)
With fever during past 48 h	46.7 (24.8–69.9)	95.7 (79–99.2)	46.7 (24.8–69.9)	100 (85.7–1)
Without fever	10.1 (5.2–18.7)	99.3 (97.6–99.8)	12.7 (7–21.8)	100 (98.8–1)

## Discussion

This study about malaria in illegal gold miners in French Guiana showed a high prevalence of *Plasmodium* spp. carriers, from 3.8 to 46.4 %, of whom 84 % were asymptomatic. Thus, overall, 18.7 % of the study population were asymptomatic malaria carriers.

### An important malaria reservoir comparable to high transmission areas…

In South America, malaria incidence has decreased in almost all transmission areas since 2000 [1]. Now, many countries aim at malaria elimination. However, *Plasmodium* spp. prevalence observed by PCR in gold miners in French Guiana is much higher nowadays than in populations from other South American countries: 3 % in the Brazilian Amazon in 2013 [20], 3.9 % for *P. vivax,* 6.7 % for *P. falciparum* in Peru in 2015 [21], and 5.8–16.5 % in Colombia in 2013 (asymptomatic carriers) [22]. Moreover, despite the fact that malaria epidemiology on the Guiana Shield is often compared to Southeast Asia [17], the present results were very high compared to the 2.1 and 5.4 % observed in Thailand in 2012 [23] and on the Cambodian border in 2015 [24], respectively. Considering only asymptomatic carriers, the prevalence was 4.9 % in Cambodia in 2013 [25], 3.4 % in Myanmar in 2014 [26] and up to 30.4 % in Bangladesh during the monsoon in 2014 [27]. In a meta-analysis published in 2013 [28], asymptomatic malaria prevalence ranged from 0 to 82 % by PCR throughout the world. Eleven out of 39 studies reported a higher *Plasmodium* spp. prevalence than in the studied population. These concerned transmission areas in Kenya, Ghana and Congo. Malaria prevalence observed in *garimpeiros* in French Guiana reaches the high transmission levels observed in Africa, corresponding to a meso-endemic level of transmission.

The heterogeneity of malaria prevalence among the different mining sites highlights the main hotspot of transmission in the Maripa Soula region but all mining sites have some degree of malaria prevalence. The reasons for this difference remain unclear. Entomological investigations on the distribution of *Anopheles* species present on each site may bring some clues. However, military operations, including mining camp destruction, urge people to move between different mining sites, which could raise malaria prevalence in new areas. It is possible that those observations reflect a situation that might change in time according to human migrations.

### …including a large proportion of falciparum cases for South America…

In this study, *P. falciparum* was predominant (48 vs 37 %, +10 % *P. falciparum/P. vivax*). This proportion is very high compared to the general frequency of *Plasmodium* species in French Guiana (33 % *P. falciparum* vs 66 % *P. vivax*) [29]. It is also very high when compared to the figures observed in Brazil and in South America in general, where *P. vivax* mostly predominates. But it reflects the same picture than in gold mining area on the Guiana Shield [1, 11].

### …with a high transmission potential…

There is no standard definition of asymptomatic parasitaemia. Many studies considered having no measurable fever at time of inclusion [28], some also required an absence of fever during follow-up, others used different clinical symptoms [20, 30], or excluded people having had anti-malarial treatment before inclusion [27]. Here it was the absence of fever during the last 48 h.

Two main ideas could explain asymptomatic carriage. First, a previous use of anti-malarial drugs can lead to the persistence of parasites at low density, particularly when the treatment course had been inadequate and/or the anti-malarials taken were of poor quality [31]. Second, in areas of high transmission, the exposure to different genetically distinct parasite sub-populations leads to the development of partial immunity allowing asymptomatic parasitaemia [28, 32]. In this study, 17 % of asymptomatic carriers had taken anti-malarials in the past month. Therefore, acquired immunity could explain the 83 % of asymptomatic carriers.

Capability of malaria transmission implies the presence of gametocytes in the blood. In this study, the PCR used did not allow differentiation of sexual from asexual forms, but several studies showed that asymptomatic persons carry gametocytes [20, 28, 32]. Even if the gametocyte density remains unclear, studies showed that asymptomatic patients can infect mosquitoes with an infection rate of 1.2, versus 22 % for symptomatic carriers, in the Amazon basin [33]. A meta-analysis in Africa in 2012 showed that 27.6 % of individuals with sub-microscopic malaria were able to infect mosquitoes, with a mosquito infection rate of 5 % [34]. A 1-year, follow-up study of 347 persons in Ghana using genotyping (to differentiate from re-infection) showed that *P. falciparum* persisted in the circulation for 194 days on average [35]. Therefore, even if the rate of infection to mosquito is lower with asymptomatic carriers, they carry gametocytes longer than symptomatic patients [26], and could therefore be a major contributor to malaria transmission. The high proportion of asymptomatic carriers in *garimpeiros* in French Guiana is a threat for transmission of malaria to the neighbouring population on the Guiana Shield and beyond.

### …and a potential individual risk

In addition to this public health issue, what is the personal risk of being a *Plasmodium* spp. carrier? Few studies have followed the clinical evolution of asymptomatic carriers. Differentiating new from asymptomatic infections becoming symptomatic is difficult [28]. A follow-up of patients with genotyping would be required. Without differentiating both, a study in Brazil showed that 17% of asymptomatic carriers became symptomatic within a 6-week follow-up [20].

### Implication for public health in French Guiana (France) and in its proximity

Considering the prevalence found in this study and the estimation of the gold-miner population (between 10,000 and 15,000), 1830–3945 persons might be carrying malaria parasites in the forest at a given time. Although the data from the symptomatic cases recorded in the health surveillance system are reassuring, those results suggest that they account for only the tip of the malaria ‘iceberg’. This study demonstrates that *garimpeiros* in French Guiana are a huge malaria reservoir with a high transmission potential. This leads to two major public health issues: (1) the improper self-medication to treat malaria symptoms raises the threat of artemisinin resistance emergence [17]; and, (2) the high mobility of this population may increase the risk of malaria spreading on the Guiana Shield and beyond, and puts local populations at risk of new malaria outbreaks.

Public health action towards asymptomatic carriers is possible. Massive drug administration (MDA), massive screening and treatment (MSaT) and focused screening and treatment (FSaT) are currently being evaluated throughout the world using molecular tools such as loop-mediated iso-thermal amplification (LAMP) [28, 32, 36, 37]. Far from elimination strategies, French Guiana is on the step of control strategy: track, test, treat (T3), but no implementation of T3 strategy at illegal gold-mining sites is programmed. Moreover, the monodose of primaquine, recommended by WHO to reduce transmission, is not implemented in France due to administrative complexities.

### Latitude for public health actions

Malaria in France concerns only French Guiana and Mayotte, a French island in the Indian Ocean, which has a completely different context. No French national plan to control malaria exists. Since the amendment of French health authorities in 2010, Regional Health Agencies (RHAs) have the mandate to implement health policies in their territories. In April 2015, a malaria control strategy was elaborated by the RHA of French Guiana with the support of several malaria experts. This 2015–2018 plan targets a switch to a pre-elimination phase in 2018. However, concerns about malaria at gold-mining sites are poorly addressed and is mainly limited to evaluating the situation [38]. This gap has been justified by: (1) the complexity to access undocumented populations conducting illegal activities in the Amazonian natural reserve; (2) safety concerns at mining sites; (3) the interdiction to diagnose and treat malaria by non-medical doctors in France; and, (4) public opinion against mining activities, and unpopularity of investing in miners’ health. The seriousness of the situation is demonstrated by these points and the issue of malaria transmission among gold miners should be addressed.

### Potential public health intervention depending on political will

Several strategies have been discussed for several years at different levels: (1) locally: by the RHA, the prefect (regional state representative), researchers, the French Army Health Department, and healthcare structures; (2) nationally: by the Direction Générale de la Santé, the Ministry of Foreign Affairs, the Home Affairs Ministry, the Overseas Territories Ministry; and, (3) internationally: by representatives of Brazil and Suriname, WHO and Pan-American Health Organization (PAHO), and the Global Fund.

In a first stage, control strategies: “test, treat and track”, provision of impregnated bed nets, and information about the importance of completing treatment could decrease local transmission in mining areas. Innovative strategies have been suggested. Malaria kits for self-diagnosis and treatment could be distributed after a training in resting sites, in collaboration with Suriname and Brazil. These places along the borders are easily accessible and appropriate for implementing public health interventions among *garimpeiros*, as they have time off and health structures are already in place (malaria clinics in Surinam).

In a second phase, when the local transmission will be lower, further strategies could target asymptomatic carriers in order to avoid malaria spread in the region and in mining sites with low transmission. For example, MDA could target people working in the most infected areas; or the use of LAMP would allow the detection and treatment of asymptomatic carriers.

In July 2015, the French Minister of Health committed to work towards malaria elimination [39], but the practical steps to do this have yet to be taken.

In this trans-border context, the fight against malaria needs a regional approach with involvement of neighbouring Suriname and Brazil, and France can no longer ignore the reality in its territory because the evidence is there: 22.3 % of gold miners carried malaria parasites in the French Guianan forest. A public health response is urgently required.

## Conclusions

The prevalence of *Plasmodium* spp. carriers detected by nested-PCR in illegal gold miners working in French Guiana was very high, 22.3 %, of which 84 % were asymptomatic. Ignoring the burden of the disease in this neglected and mobile population may increase not only the spread of malaria on the Guiana Shield and beyond, but also the risk of emergence of artemisinin resistance, a global threat.

## Abbreviations

ACT: artemisinin-based combination therapy
CI: confidence interval
FSaT: focused screening and treating
IR: interquartile range
LAMP: loop-mediated iso-thermal amplification
MDA: massive drug administration
MSaT: massive screening and treating
PCR: polymerase chain reaction
RDT: rapid diagnostic test
WHO: World Health Organization
